# Codon Optimization of the Human Papillomavirus E7 Oncogene Induces a CD8^+^ T Cell Response to a Cryptic Epitope Not Harbored by Wild-Type E7

**DOI:** 10.1371/journal.pone.0121633

**Published:** 2015-03-23

**Authors:** Felix K. M. Lorenz, Susanne Wilde, Katrin Voigt, Elisa Kieback, Barbara Mosetter, Dolores J. Schendel, Wolfgang Uckert

**Affiliations:** 1 Max-Delbrück-Center for Molecular Medicine, Berlin, Germany; 2 Institute for Molecular Immunology, Helmholtz-Zentrum München, Munich, Germany; 3 Institute of Biology, Humboldt University, Berlin, Germany; Maisonneuve-Rosemont Hospital, CANADA

## Abstract

Codon optimization of nucleotide sequences is a widely used method to achieve high levels of transgene expression for basic and clinical research. Until now, immunological side effects have not been described. To trigger T cell responses against human papillomavirus, we incubated T cells with dendritic cells that were pulsed with RNA encoding the codon-optimized E7 oncogene. All T cell receptors isolated from responding T cell clones recognized target cells expressing the codon-optimized E7 gene but not the wild type E7 sequence. Epitope mapping revealed recognition of a cryptic epitope from the +3 alternative reading frame of codon-optimized E7, which is not encoded by the wild type E7 sequence. The introduction of a stop codon into the +3 alternative reading frame protected the transgene product from recognition by T cell receptor gene-modified T cells. This is the first experimental study demonstrating that codon optimization can render a transgene artificially immunogenic through generation of a dominant cryptic epitope. This finding may be of great importance for the clinical field of gene therapy to avoid rejection of gene-corrected cells and for the design of DNA- and RNA-based vaccines, where codon optimization may artificially add a strong immunogenic component to the vaccine.

## Introduction

The expression of sufficient amounts of transgenic protein in a gene-modified cell is crucial in molecular biology and clinical biotechnology. Since gene synthesis has become a time- and cost-efficient method for the design of nucleotide sequences, codon optimization has been established as a standard tool to maximize protein expression in a desired system. The genetic code for translating nucleotide sequences to proteins uses 64 nucleotide triplets (codons), which encode 20 amino acids and three translational stop signals. Through this degenerated code certain amino acids are encoded by up to six synonymous codons [1]. The frequencies of different tRNAs loaded with the same amino acid to elongate the nascent protein chain from the ribosome vary and are species-specific [2]. Replacement of unfavorable codons with low tRNA frequency, adaption of GC content, avoidance of repetitive sequences and unwanted mRNA secondary structures are key modifications introduced by codon-optimization algorithms to achieve up to 1000-fold higher expression levels of a protein [3].

Clinical and pharmaceutical research has focused on adapting transgene sequences to host cell systems using codon optimization. It has been shown that codon optimization of transgene cassettes enhances efficacy in preclinical models of gene correction therapy and clinical trials, where long-term compensation for the lack of functional endogenous protein is desired [4–7]. A second growing field in which codon optimization has been beneficial is the development of DNA vaccines. Sufficient expression of a gene in antigen-presenting cells, e.g. via codon optimization, is key to induce protective immune responses against target pathogens after vaccination [8–12]. Furthermore, some cancer vaccination strategies use dendritic cells (DCs) that have been transfected with *in vitro*-transcribed RNA (ivtRNA) encoding a codon-optimized transgene to prime the immune system against viral or tumor-associated antigens [13].

However, it remains to be investigated what immunological influence codon-optimization has on cells. It has been described that cryptic epitopes from alternative reading frames (ARFs) can be a source of T cell epitopes [14,15], but thus far experimental data demonstrating the generation of cryptic epitopes from codon-optimized gene sequences are lacking.

In this study, we attempted to isolate human papillomavirus (HPV) E7-specific T cell receptors (TCR) for TCR gene therapy. HPV accounts for more than 99% of cervical cancers worldwide, of which more than 50% are positive for HPV16 [16,17]. HPV16 oncogene E7 has been proposed as an ideal target for immunotherapy of cervical cancer [18,19]. One evolutionary escape mechanism of HPV is to keep its transcriptional activity low through the usage of inefficient codons that lead to weak expression of E7 protein in human cells to reduce recognition by the immune system [20]. Thus, it has been proposed that codon-optimization of HPV oncogenes could be used to increase expression in target cells for enhanced immunogenicity to isolate E7-specific TCRs [21–23].

Here, we used DCs as professional antigen presenting cells expressing HPV16 E7 from codon-optimized ivtRNA to screen an autologous T cell repertoire for E7-specific T cells. Codon-optimized E7 (E7co) indeed allowed us to achieve high expression levels of E7 protein in DCs and enabled efficient T cell priming. We identified and cloned antigen-specific TCR genes and expressed them via a retroviral vector in peripheral blood mononuclear cells (PBMCs) for downstream MHC restriction analysis and mapping of antigenic epitopes within the E7 gene. Surprisingly, all candidate TCRs were highly specific for an epitope of the +3 ARF of E7co, which is not translated from the natural E7wt sequence. To our knowledge this is the first study reporting that codon-optimization can render a transgene artificially immunogenic.

## Results

### Codon optimization increases E7 expression

**Fig 1:**
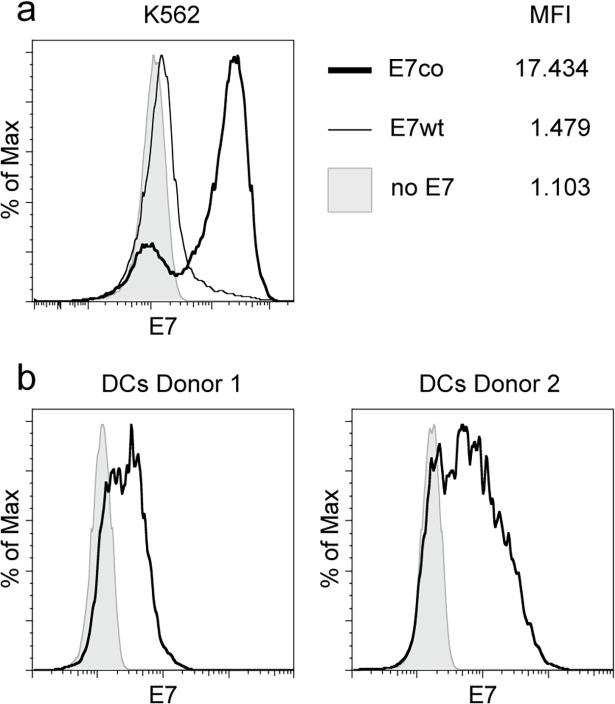
Expression of HPV16 E7 from codon-optimized (co) and wild type (wt) sequences. (a) The myelogenous leukemia cell line K562 was transfected via electroporation with 15 μg of E7wt (thin line) or E7co (bold line) ivtRNA, stained intracellularly for E7 protein expression, analyzed by flow cytometry and depicted on a bi-exponential scale of a histogram. Non-electroporated cells (gray area) serve as negative control. MFI (median fluorescence intensity) values have been calculated by FlowJo8.7. (b) DCs from two healthy donors were electroporated with E7co ivtRNA and E7 protein expression was confirmed by flow cytometric analysis (bold line). Non-electroporated DCs (gray area) were used as a control.

We generated ivtRNA from plasmids encoding HPV16 E7wt and E7co, which were used to electroporate K562 cells [24] to compare expression levels. Intracellular staining of E7 followed by flow cytometric analysis confirmed that inefficient codon usage in the E7wt gene sequence led to weak expression [20]. In contrast, E7 expression from codon-optimized ivtRNA improved intracellular protein expression by increasing the mean fluorescence intensity (MFI) approximately 12-fold compared to E7wt-expressing cells (Fig. 1A).

### Generation of E7co-specific T cell clones and cloning of candidate TCRs

Mature DCs were generated from plate-adherent monocytes and transfected via electroporation with E7co ivtRNA for translation, processing and cell surface presentation of the transgene epitopes [25]. FACS analysis showed that E7 protein was expressed by the DCs (Fig. 1B). Autologous PBMCs were enriched for CD8^+^ T cells via magnetic beads (S1A Fig.) and incubated with E7co-expressing DCs. T cells were expanded by two rounds of stimulation and enriched for the T cell activation marker CD137 [26] (S1B Fig.). Variations in CD137 enrichment are due to different separation efficiencies. However, this did not affect the generation of T cell clones, which recognized E7co-expressing target cells. We selected four T cell clones showing the strongest reactivity to E7co-expressing DCs for TCR analysis (S2A Fig.). Usage of VJ alpha and VDJ beta chains and CDR3 sequences are shown in S1 Table. Two TCRs were identified from each T cell donor. All four TCRs differed in V(D)J gene usage and CDR3 regions of the alpha and beta chains showed no homology. TCRs were cloned into a retroviral expression vector to transduce T cells [27,28] for analysis of TCR properties. After transduction 30–40% of CD8^+^ T cells expressed the transgenic TCR (S2B Fig.) and were used for further MHC restriction analysis and epitope mapping.

### TCR-transduced T cells recognize E7co- but not E7wt-expressing HLA-B*27:05^+^ target cells

**Fig 2:**
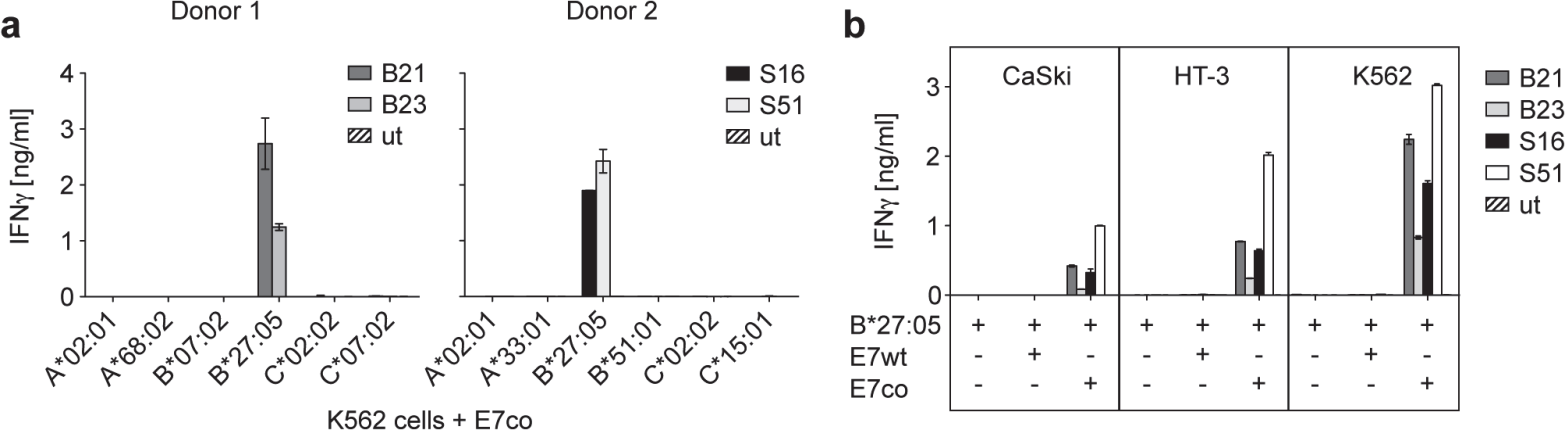
TCRs are specific for HLA-B*27:05 and E7co but not E7wt. TCR-transduced T cells were tested for HLA restriction and antigen specificity by coculture with different target cells to measure IFNγ release by ELISA. Untransduced (ut) T cells were used as negative control. Results are shown as mean +/− SEM of duplicates. (a) Restriction mapping of four different TCRs (B21, B23, S16 and S51) was perfomed using K562 target cells carrying E7co and one of the six cognate MHC class I molecules of the original donor. (b) HLA-B*27:05-engineered target cell lines of different origin (CaSki, HT-3 and K562) were tested for recognition by TCR-transduced T cells.

We used single MHC class I allele expressing K562 target cells [29,30] to identify the MHC-restriction elements that present the E7co-derived epitopes recognized by the TCRs. Therefore, single MHC target cells were stably transduced with E7co and E7wt antigen sequences. TCRs were screened for antigen reactivity in combination with one of the six cognate MHC class I molecules of the original donor. All TCR-transduced T cells recognized target cells positive for E7co and the MHC-restriction allele HLA-B*27:05, which was shared by both donors (Fig. 2A). Interestingly, all TCR-transduced T cells released high amounts of interferon-γ (IFNγ) when cocultured with cell lines that expressed E7co. In contrast, none of the TCR-transduced T cells released IFNγ upon coculture with target cells containing E7wt (Fig. 2B). Thus, we further investigated whether the TCRs were specific for an epitope generated only from E7co, or rather that lack of recognition of E7wt was due to its low expression levels.

### TCRs are specific for a peptide from a 30-nucleotide microgene fragment of E7co

**Fig 3:**
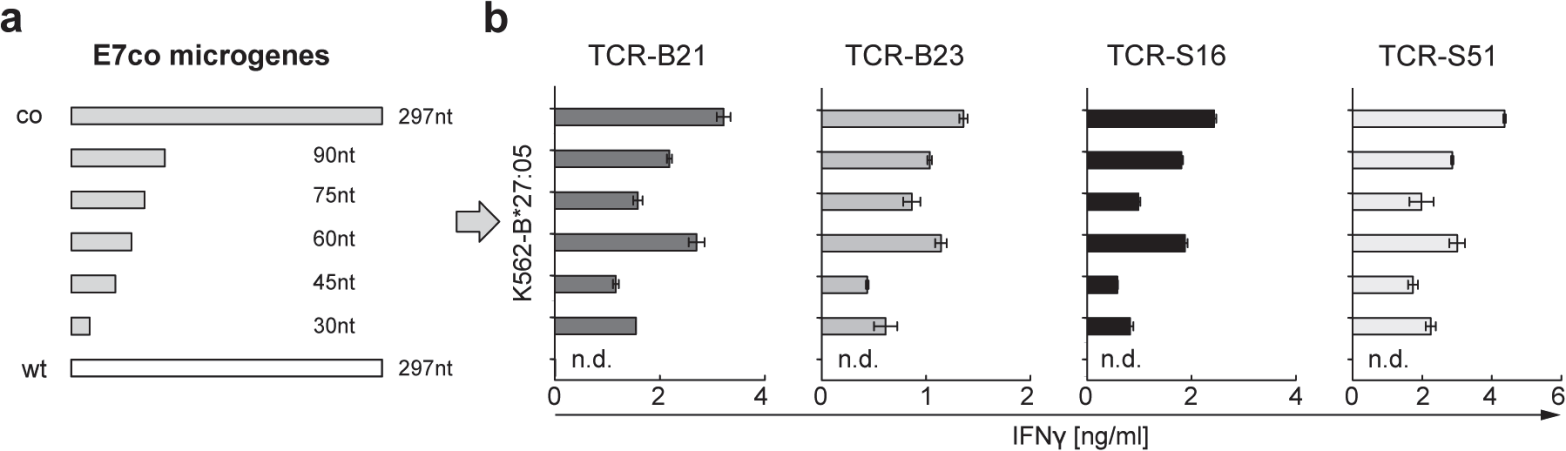
Epitope mapping with truncated E7co microgene fragments. (a) Scheme of truncated E7co microgenes (grey bars) and full-length E7wt (white bar) that were stably expressed in K562-HLA-B*27:05 target cells through MP71 retrovirus transduction. Microgenes were coupled to mCherry expression marker via an IRES element to confirm transgene expression. (b) TCR-transduced T cells (B21, B23, S16 and S51) were cocultured with microgene-expressing target cells and supernatant was tested for IFNγ by ELISA. Untransduced T cells were used as negative control. Results are shown as mean +/− SEM of duplicates. n.d., not detectable.

To narrow the gene region encoding the epitope recognized by TCR-transduced T cells, we generated truncated versions of the full-length E7co gene (minigenes in S3A Fig., microgenes in Fig. 3A) and stably expressed them via retroviral transduction in K562-B*27:05 cells. Mini- and microgenes were linked to a fluorescent marker gene (mCherry) via an internal ribosomal entry site (IRES) to confirm transgene expression by flow cytometry. All TCR-transduced T cells recognized minigenes harboring the 5’ 105 nucleotides of E7co (S3B Fig.). Further analysis with truncated microgenes showed that all T cells transduced with one of the four TCRs released IFNγ when only the 5’ 30-nucleotide microgene fragment was present in K562-B*27:05 target cells (Fig. 3B). Notably, target cells carrying E7wt never elicited IFNγ secretion by TCR-transduced T cells.

### A cryptic epitope from the +3 ARF of E7co accounts for T cell reactivity

**Fig 4:**
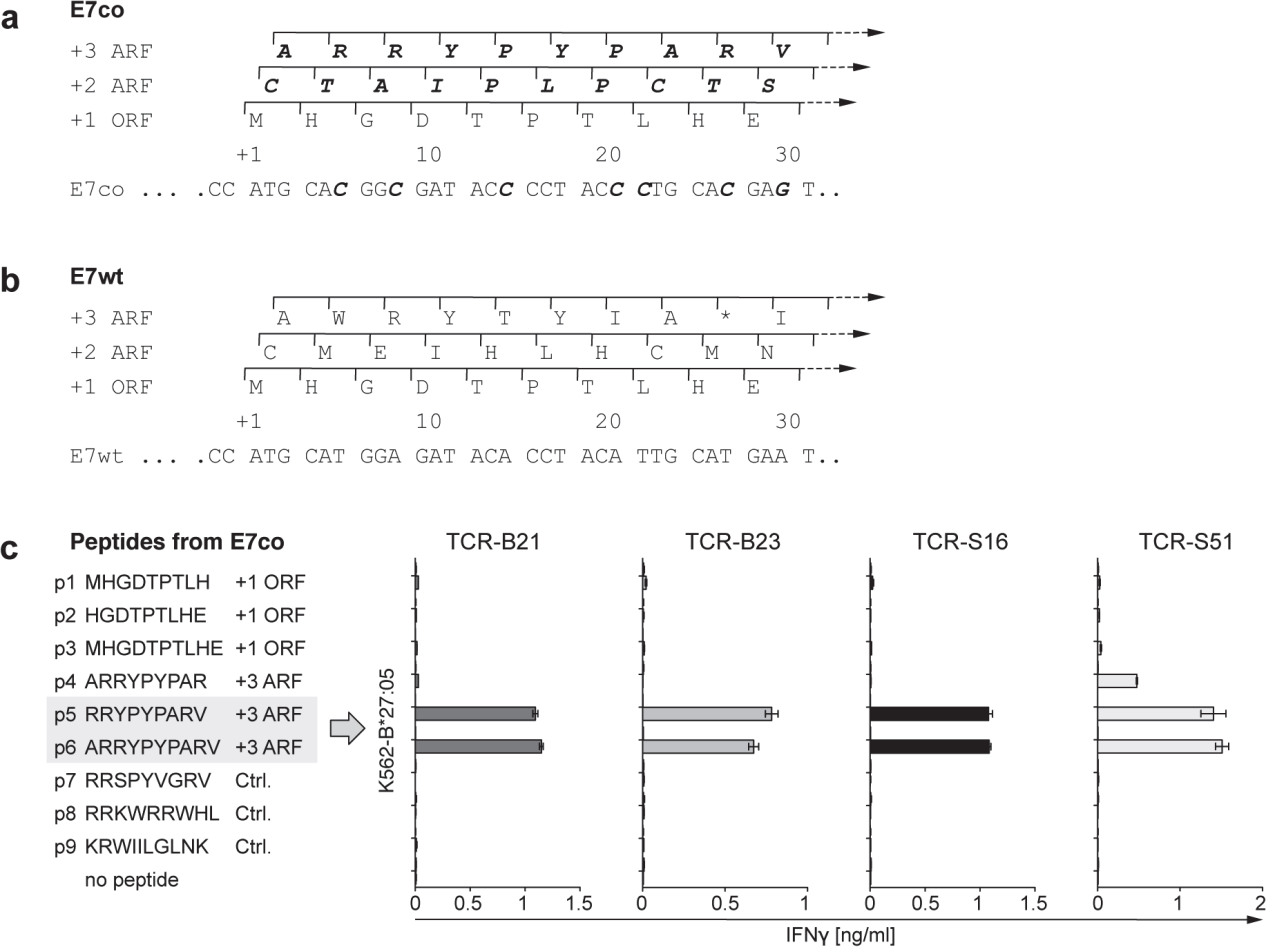
Epitope mapping identifies a cryptic epitope from +3 ARF of E7co as TCR target. (a–b) The +2 and +3 ARFs of E7co translate for cryptic peptide sequences (bold italic) not encoded by the +2 and +3 ARFs of E7wt. (c) T cells transduced with TCRs B21, B23, S16 and S51 were cocultured with K562-B*27:05 cells pulsed with candidate epitopes from the +1 ORF and the +3 ARF of E7co and a selection of control peptides (Ctrl.). Untransduced T cells were used as negative control. Reactivity was asessed by IFNγ ELISA. Results are shown as mean +/− SEM of duplicates.

Next, we performed epitope mapping to determine exact epitope specificity of the four TCRs. Identification of T cell epitopes is supported by epitope prediction algorithms that calculate *in silico* epitope binding strength to a given MHC molecule. Binding affinity of MHC class I epitopes has been proposed as a key factor for the immunogenicity of peptides [31]. Cytotoxic CD8^+^ T cells reactive against high-affinity epitopes show enhanced killing and rejection of tumors [32]. Interestingly, different sources of MHC class I epitopes have been suggested including polypeptides translated from ARFs [33]. Indeed, E7co contains three reading frames—the reference +1 open reading frame (ORF) and two unconventional open reading frames (+2 ARF, +3 ARF) (Fig. 4A). The ORF starts with the +1 adenine of the ATG/AUG start codon and encodes the primary E7 protein sequence. Starting translation one or two nucleotides downstream, the +2 ARF and the +3 ARF do not contain stop codons within the first 30 nucleotides of E7co. However, neither of these ARFs have an in-frame ATG start codon (Fig. 4A) nor any non-canonical translational initiation site (TIS) [34].

We used a prediction server (netMHCpan 2.8 [35]) to evaluate HLA-B*27:05 binding affinity of 9-mer and 10-mer peptides derived from the three reading frames of the 30-nucleotide microgene fragment of E7co to predict candidate peptides for epitope mapping. However, peptides from the +1 ORF (Table 1) and the +2 ARF were not predicted to bind to HLA-B*27:05. The algorithm predicted peptide (p5) RRYPYPARV of the E7co +3 ARF as being a strong binder to HLA-B*27:05 and thus as a potential T cell epitope. Additionally the p5 derivatives, p4 and p6, were also predicted to bind to HLA-B*27:05. It should be noted that while the +1 ORF of E7wt encodes the same primary E7 protein as the +1 ORF of E7co, the +3 ARF of E7wt harbors a stop codon, thereby not encoding any 9- or 10-mer peptide (Fig. 4B).

**Table 1:** Epitope binding prediction to HLA-B*27:05.

#	-mer	Sequence	Affinity to MHC [nM]	Binding Level	Origin
p1	9	MHGDTPTLH	27486.64	-	E7wt/co +1 ORF
p2	9	HGDTPTLHE	44005.14	-	E7wt/co +1 ORF
p3	10	MHGDTPTLHE	37485.07	-	E7wt/co +1 ORF
p4	9	ARRYPYPAR	719.27	WB	E7co +3 ARF
p5	9	RRYPYPARV	72.61	SB	E7co +3 ARF
p6	10	ARRYPYPARV	1399.45	WB	E7co +3 ARF
p7	9	RRSPYVGRV	114.27	SB	Pl. falciparum
p8	9	RRKWRRWHL	53.23	SB	self-peptide
p9	10	KRWIILGLNK	31.85	SB	HIV gag

HLA-B*27:05-binding affinities of peptides translated from the primary E7wt/co +1 ORF (p1—p3) and the +3 ARF (p4—p6) of the 30-nt microgene fragment (Fig. 4A) of E7co were determined with netMHCpan 2.8 prediction server. Epitope binding levels (SB, strong binder; WB, weak binder) were predicted by netMHCpan 2.8 according to default threshold settings of the program. Control peptides (p7—p9) with high binding affinities to HLA-B*27:05 were included for testing of TCR specificity.

Experimental confirmation of the epitope predictions was made using target K562-B*27:05 target cells, which were pulsed with peptides prior to coculture with TCR-transduced T cells. In accordance with epitope prediction, all TCR-transduced T cells released IFNγ upon coculture with target cells pulsed with the cryptic +3 ARF epitope p5 from E7co (Fig. 4C). Additionally, all TCR-transduced T cells released IFNγ upon coculture with the p6 epitope, which is a 10-mer derivate of p5. TCR S51 further recognized p4, another derivate of p5. All these epitopes share the core 8 amino acid sequence RRYPYPAR from the +3 ARF of E7co. As expected from the epitope binding predictions, epitopes encoded by the primary +1 ORF of E7wt/co were not recognized by the TCRs. Importantly, control peptides predicted to be strong HLA-B*27:05 binders (p8, p9) or possessing high sequence similarity to p5 (p7) were not recognized. These findings were reproduced using HT-3 cells as target cells following stable transduction with HLA-B*27:05 and E7co constructs and further confirmed by peptide-pulsing experiments.

In sum, the results demonstrated that all candidate TCRs isolated from antigen-specific T cells that were primed by E7co-expressing DCs, were highly specific for a cryptic epitope of the +3 ARF of E7co, a reading frame that was artificially generated by codon optimization.

### Redesign of E7co to prevent the generation of the immunogenic cryptic epitope

**Fig 5:**
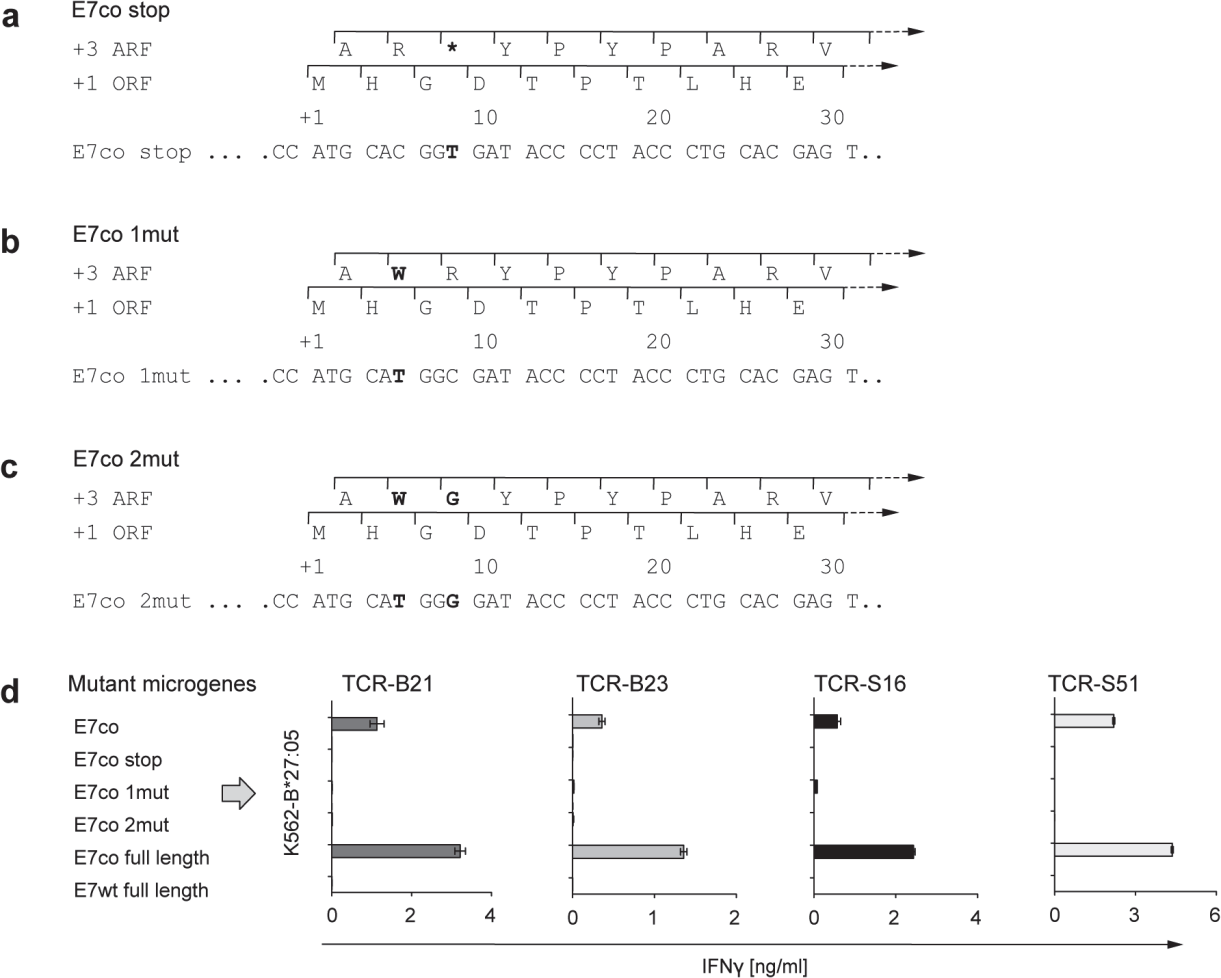
Redesign of +3 ARF prevents E7co from recognition by TCR-transduced T cells. (a–c) Exchanging wobble nucleotides (bold) of the primary +1 ORF leads to (a) introduction of stop codons or (b–c) amino acid exchanges (1mut, 2mut) at anchor residue positions only in the +3 ARF (bold amino acids) without changing the polypeptide sequence from +1 ORF translation. (d) TCR-tranduced T cells (B21, B23, S16 and S51) were cocultured with K562-B*27:05 target cells that express one of the mutant microgene constructs (1–30 nt) of E7co. Untransduced T cells were used as a negative control. Reactivity was assessed by IFNγ ELISA. Results are shown as mean +/− SEM of duplicates.

For certain applications it is crucial to achieve high gene expression levels using codon optimization. Thus, we sought to develop a strategy to introduce a point mutation in the E7co sequence to abrogate the expression of the immunogenic cryptic epitope p5, without disturbing the expression of the primary +1 ORF. Mutation of adenine at position 9 to thymidine (A9T) in the +1 ORF exchanges a wobble nucleotide whereby the degenerate code allows the translation of glycine from GGA as well as from GGT. In the +3 ARF this A9T nucleotide exchange led to a codon switch from AGA (arginine) to TGA (stop) and hence to the disruption of the +3 ARF (Fig. 5A). Moreover, we determined if anchor residue modifications of the cryptic epitope p5 at peptide positions 1 and 2 influenced MHC class I binding and recognition by the TCRs. Therefore, we mutated cytidine to thymidine at nucleotide position 6 (C6T), leading to an arginine to tryptophan exchange in the +3 ARF (Fig. 5B). Furthermore, we constructed an E7co mutant carrying the C6T mutation and additionally a C9G mutation mediating an arginine to glycine exchange at anchor residue position 2 of peptide p5 (Fig. 5C). This mutation in the +3 ARF does not affect amino acid translation from the primary +1 ORF. All constructs were generated as 30-nucleotide microgene fragments and stably introduced into K562-B*27:05 target cells by retroviral transduction. Expression of the fragments was controlled by fluorescent marker gene expression.

While all TCR-transduced T cells were reactive to the unmodified 30-nucleotide microgene fragment of E7co, none of the TCR-transduced T cells released IFNγ when cocultured with K562-HLA-B*27:05 harboring one of the mutated microgene constructs, where the +3 ARF was modified (Fig. 5D). Target recognition of TCRs in this assay followed a strict on-off pattern. The exchange of only one nucleotide of E7co abrogated target cell recognition by all four TCR-transduced T cells, showing their exquisite specificity for the cryptic epitope.

## Discussion

Codon optimization has become a readily available tool to increase the expression of transgenes in basic research as well as clinical settings, but codon optimization may also affect polypeptide sequences translated from the +2 and +3 ARFs. Cryptic frame-shift epitopes have been shown to be immunogenic components of a gene sequence [14,15,36], however, the generation of cryptic epitopes from codon-optimized gene sequences has not been described yet. In this study, we report that codon-optimization renders the HPV16 E7 oncogene artificially immunogenic via the generation of a cryptic epitope from an ARF, which does not exist in the wild type sequence. We isolated TCRs from all antigen-specific candidate T cell clones that were stimulated with DCs expressing the E7 antigen using a codon-optimized gene sequence. Unexpectedly, all TCRs were highly specific for a cryptic epitope translated from the +3 ARF of E7co. None of the TCRs isolated was specific for the primary E7 oncoprotein. Importantly, this cryptic epitope was not encoded by the +3 ARF of the wild type gene sequence, thus making it a strong immunogenic component of the codon-optimized transgene, which does not reflect the natural E7wt sequence.

In the initial attempt to generate T cells specific for HPV16 E7, we used DCs pulsed with E7co ivtRNA as professional antigen-presenting cells to allow only naturally processed epitopes to be presented on the cell surface. However, the generation of a cryptic frame-shift epitope is not a specific attribute of the DCs, because all epitope-specific TCRs that could be identified also recognized K562, CaSki and HT-3 target cell lines that were engineered to express HLA-B*27:05 and E7co. Thus, the generation of the frame-shift epitope seems to be a general property of primary human cells and cell lines [33].

Several non-canonical sites for initiation of translation have been described that provide alternative routes of polypeptide expression [34]. In our expression system we did not find an alternative start codon in the 30-nucleotide microgene of E7co or an ATG start codon in the 5’ untranslated region (5’ UTR). Also, the translation of the cryptic peptide from the +3 ARF was independent of the vector backbone used to introduce E7co into target cells via a retroviral vector or ivtRNA. Although both vectors—the MP71 retroviral vector for stable transduction and the pcDNA3.1 vector for T7-promoted ivtRNA generation—differ in the 5’ UTR, the target cells were always recognized, thereby excluding a specific effect of the 5’ UTR in influencing frame-shift translations in these cases.

The question remains how the cryptic epitope was generated. One mechanism that could account for our observations is the generation of DRiPs (defective ribosomal product) [37,38]. DRiPs are short polypeptides that may be generated from ARFs in the 5’ region of a gene. Additionally, ribosomal scanning for ARFs, doublet decoding [39] or simply translational initiation “noise” may account for the generation of the p5 cryptic epitope. Biologically, all these potential sources of epitopes reflect a cell’s translationally active nucleotide sequences. This provides a fast response to changes in the cell, for example through somatic mutations or the introduction of foreign nucleotide sequences, which can be monitored by the immune system through changes of the MHC class I epitope repertoire at the cell surface [15]. In our example, the generation of a polypeptide from the +3 ARF led to the processing and presentation of p5 epitope with high binding affinity to HLA-B*27:05, which is a critical parameter for eliciting a strong T cell response [32]. This epitope is an artificial epitope that has no homologue in humans or other organisms, as confirmed by BLAST search. Therefore, the T cell repertoire against this peptide was not tolerized in the donors, allowing strong T cell responses.

One important aspect that could influence the immunological consequences of codon optimization is the applied algorithm provided by different companies or software programs. We compared five different codon optimization results for HPV16 E7wt and predicted the HLA-B*27:05 binding affinities of the resulting peptides from the +3 ARFs with the netMHCpan 2.8 prediction server. None of the algorithms automatically introduced a stop codon in one of the ARFs to disrupt possible polypeptide translation. Furthermore, although all polypeptide sequences translated from the +3 ARFs of the different codon-optimized E7 genes differed by one to three amino acids, all sequences contained epitopes with high binding affinities to HLA-B*27:05 according to the netMHCpan 2.8 prediction server since they all had the same critical HLA-B*27:05-binding anchor residues (e.g. R at position 2) [40]. Therefore, epitopes from the +3 ARF of all codon-optimized E7 sequences may have had the potential to elicit a T cell response.

While we identified a cryptic epitope derived from HPV16 E7co binding to HLA-B*27:05 in this study, in theory every codon-optimized sequence may carry cryptic neo-epitopes translated from ARFs that bind to one of the many highly polymorphic MHC alleles. Thus, it is desirable to combine optimization algorithms with MHC class I epitope prediction to avoid the translation of potentially immunogenic neo-epitopes. The induction of unwanted immune responses to cryptic epitopes could have severe consequences for clinical application of codon-optimized transgenes.

To improve the therapeutic efficacy of DNA vaccines, codon optimization has been described to increase the immunogenicity of the transferred sequence [41]. However, in our setting the expression of an immunogenic frame-shift epitope from an ARF led to strong cellular immune responses to this cryptic epitope, without an immune response to the desired primary E7 protein. This may be due to an immunodominant effect of the cryptic epitope over other epitopes [42], although strong immune responses to the E7 protein are generally rare. Such an immunodominant effect would be a serious drawback of a codon-optimized DNA vaccine hampering immune responses to the primary target protein. The expression of a cryptic frame-shift epitope may also occur when using DC vaccines, which were transfected with codon-optimized ivtRNA encoding viral or tumor-associated antigens.

One further clinical setting where codon-optimization of the transgene has been shown to increase therapeutic efficacy, is classical gene correction therapy [4–7]. In this case the expression of only one cryptic frame-shift epitope that allows MHC-presention and recognition as a foreign epitope by a patient’s T cell repertoire may render gene-corrected cells prone to T cell attack and rejection [14]. Depending on the individual MHC constitution of a patient, rejection may only occur in those patients with MHC class I alleles that have a high binding affinity for the cryptic epitope.

In conclusion, we describe here that codon optimization, which is usually applied to increase the level of gene expression, added an unwanted immunogenic factor to the HPV16 E7 transgene. In general, we highly suggest considering this observation for a variety of clinical applications using codon-optimized transgenes. If experimental or clinical success depends on increased gene expression through codon optimization, our findings reveal the importance of introducing stop codons or mutations in the 5’ region of ARFs without affecting translation from the primary +1 ORF to avoid translation of immunogenic cryptic peptides or to disrupt the binding of mutated cryptic peptides to a patient’s MHC molecule.

## Material and Methods

### Codon optimization and production of ivtRNA

HPV16 E7wt reference sequence [43] was codon-optimized and synthesized at GeneArt (Life Technologies, Carlsbad, CA). E7wt derived from cDNA of CaSki cells (ATCC, Manassas, VA) and E7co were cloned into the pcDNA3.1(-) vector (Invitrogen, Life Technologies) via NotI and EcoRI restriction sites. ivtRNA was generated with the T7 promoter-driven mMESSAGE mMACHINE Kit (Ambion, Life Technologies) and a poly-A tail was added to the ivtRNA using the Poly(A) tailing kit (Ambion). Target cells were transfected via electroporation with 15–25 μg E7co ivtRNA as described [44]. Alternatively, codon optimization of E7wt was assessed *in silico* by GeneArt, GenScript (Piscataway, NJ), GeneWiz (South Plainfield, NJ), IDT (Integrated DNA Technologies, Coralville, IA) and JCat (Java Codon Adaption Tool, Braunschweig, Germany) algorithms.

### Generation of E7co-specific T cell clones

Blood samples were drawn with written and informed consent of donors. The institutional review board of the University Hospital of the Ludwig-Maximilians-University Munich approved, according to national law, to take blood from healthy donors for this study (Principles and application of adoptive T cell therapy; ethics committee project number 071-06–075-06). Autologous mature DCs were generated from plate adherent monocytes with an 8d protocol (donor 1) [44]. For donor 2, an optimized protocol was applied, which enabled the efficient generation of immunostimulatory mature DCs in 3d [45]. Stimulation of T cells with autologous DCs was performed as described [46]. In brief, DCs were pulsed with 25 μg E7co ivtRNA. Four to six hours after electroporation, 1x10^6^ DCs (8d) or 2x10^5^ DCs (3d) were cocultured with 1x10^6^ CD8^+^ T cells isolated from T cells via negative selection using the CD8^+^ T Cell Isolation Kit II (Miltenyi, Bergisch Gladbach, Germany) in T cell medium (RPMI1640, Gibco, Life Technologies; 10% heat-inactivated human serum, 4 mM L-glutamine, 12.5 mM HEPES, 100 U/ml penicillin/streptomycin). IL-7 (5 ng/ml; Promokine, Heidelberg, Germany) was added on day 0 and IL-2 (50 U/ml, Chiron Behring, Marburg, Germany) was added from day 2 on thrice weekly. After seven days, T cells were restimulated with newly generated ivtRNA-pulsed DCs. Fourteen days after restimulation, T cells were cocultured with ivtRNA-pulsed DCs for 12h and stained with a CD137-specific monoclonal antibody (mAb) (BD, Franklin Lakes, NJ). CD137^+^ cells were enriched using MACS magnetic beads (Miltenyi) and single T cell clones were plated in wells of a 96-well plate (TPP, Trasadingen, Switzerland) via limiting dilution. T cell clones were cultured and restimulated every 14 days as described [25]. At day 13 of a restimulation cycle 100 μl (of 200 μl per 96-well) of each T cell clone was cocultured overnight with 1-4x10^4^ of either E7co ivtRNA-pulsed DCs or H_2_O-pulsed DCs, respectively. Antigen-specific T cell clones were identified by measuring IFNγ concentration in the supernatant of the coculture with standard enzyme-linked immunosorbant assay (OptEIA Human IFN-γ ELISA Kit, BD).

### TCR identification and retroviral TCR transduction

PCR was employed to determine the TCR chains from the cDNA of E7co antigen-specific T cell clones. A panel of TCR variable region (TRAV, TRBV)-specific forward primers was combined with a TCR constant region (TRAC, TRBC)-specific reverse primer [47]. PCR-amplified products were separated via agarose gel electrophoresis, sequenced and analyzed with the IMGT/V-quest [48] server. TRAC and TRBC were exchanged by murine counterparts to increase TCR expression levels [49] and human/mouse hybrid alpha and beta chains were linked via a P2A peptide linker [27]. Codon-optimized TCR cassettes (GeneArt) [50] were cloned into the MP71-PRE vector [28] via NotI and EcoRI restriction sites. TCR vector plasmids were cotransfected with MLV gag/pol and MLV-10A1 plasmids into 293T cells for production of retroviral particles as described [51]. T cell containing PBMCs were isolated via ficoll gradient centrifugation (Biocoll, Biochrom, Berlin, Germany) from healthy donors and stimulated in CD3- (5 μg/ml OKT-3, Pharmingen, Hamburg, Germany) and CD28- (1 μg/ml, Pharmingen) mAb-coated 24-well plates (TPP) and retroviral TCR-transduction was performed as described [27]. TCR transduction efficiency was measured by staining T cells with a mAb against the transgenic murine TRBC (clone H57–597, Biolegend, San Diego, CA). For testing the specificity of the TCR, 5x10^4^ TCR-transduced T cells were cocultured overnight with 5x10^4^ K562, HT-3 or CaSki target cells (all from ATCC) in cell culture medium (RPMI1640 + GlutaMax, +1x penicillin/streptamycin, +1x MEM and + 1x sodium pyruvate, Gibco; +10% FCS, Pan Biotech, Aidenbach, Germany). IFNγ release to the supernatant was measured via ELISA as described above.

### Antigen constructs and cell culture

The HLA status of the donors was determined at the Zentrum fuer Humangenetik und Laboratoriumsdiagnostik (MVZ, Martinsried, Germany). Based upon this, single MHC-expressing K562 target cells [29,30] were used to perform restriction and epitope mapping. Therefore, we constructed 3’ truncated versions of E7co termed mini- and microgenes by PCR amplification of the desired gene region, cloned them into the MP71-PRE [28] retroviral vector plasmid via a PmlI restriction site upstream of the reporter gene mCherry linked to an IRES site. Mutated versions of the 30-nucleotide microgene were generated using primers that introduced point mutations as depicted in Fig. 5. Target cell line transduction with antigen constructs was performed by seeding 2-5x10^5^ K562, HT-3 cells or CaSki in 1 ml cell culture medium in 24-well plates the day before transduction. Generation of retrovirus-containing supernatant was carried out as described above. One ml of retrovirus-containing supernatant was added to the cells at the day of transduction followed by two hours of centrifugation at 800 g at 32°C. Adherent HT-3 and CaSki cell lines were passaged once per week by washing 2x with PBS and incubation with 0.125% trypsin-EDTA (Gibco) for 5 min at 37°C.

## Supporting Information

S1 Fig Enrichment of CD137^+^ T cells after stimulation with E7co expressing DCs. Data from two healthy donors are shown. (a) PBMCs were isolated from fresh blood via ficoll gradient centrifugation and enriched for CD8^+^ T cells by magnetic bead separation. Flow cytometry analysis of CD8^+^/CD3^+^ cells before and after CD8^+^ enrichment is shown. (b) After priming and expanding CD8^+^ T cells were stimulated with E7co-expressing DCs (E7co stim.) and sorted for CD137 activation marker via magnetic bead separation to obtain antigen-specific T cells (CD137-enriched).
(PDF)

S2 Fig Screening for antigen-specific T cell clones and retroviral expression of candidate TCRs in PBLs. (a) T cell clones of both donors were cocultured with autologous DCs electroporated with or without E7co ivtRNA to identify antigen-specific T cells via IFNγ release. Arrows indicate selected antigen-specific T cell clones for isolation of TCR genes. Cocultures were performed in duplicates depending on the amount of cells available. Duplicates are shown as mean +/− SEM. (b) TCR genes of candidate T cell clones were isolated (S1 Table) and cloned with murine constant TCR regions into retroviral vectors for efficient expression of transgenic TCR to further analyze properties of TCR gene-modified T cells. Expression of transduced TCRs in T cells was detected by staining with an antibody specific for the murine constant beta region followed by flow cytometric analysis. Results are representative for 3 independent TCR transduction experiments.
(PDF)

S3 Fig TCR-transduced T cells detect a 105-nt minigene of E7co. (a) Scheme of truncated minigenes of E7co for epitope mapping. Minigenes were stably expressed in K562-B*27:05 target cells via MP71 retrovirus transduction. Minigenes were coupled to mCherry expression marker via an IRES element to confirm transgene expression. (b) Supernatant of TCR-transduced T cells cocultured with target cells was screened for IFNγ release via ELISA. Results are shown as mean +/− SEM of duplicates.
(PDF)

S1 Table TCR VDJ-gene usage according to IMGT nomenclature. TCR VDJ-gene usage was determined by PCR from cDNA of T cell clones with TCR chain specific primer panels. Resulting sequences were analyzed with IMGT/V-quest. TRAV, T cell receptor alpha variable region; TRBV, T cell receptor beta variable region; CDR3, complementary determining region 3.
(PDF)
