# Supplementary figures and images for: Codon Optimization of the Human Papillomavirus E7 Oncogene Induces a CD8+ T Cell Response to a Cryptic Epitope Not Harbored by Wild-Type E7
Source: PLoS One. 2015 Mar 23;10(3):e0121633. doi: 10.1371/journal.pone.0121633 (PMC4370481; doi:10.1371/journal.pone.0121633)

**a****Donor 1**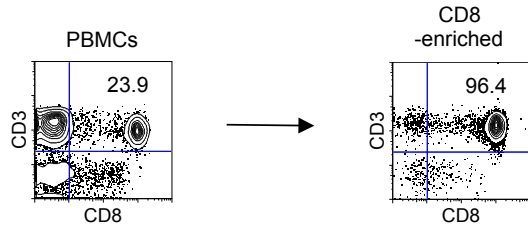**Donor 2**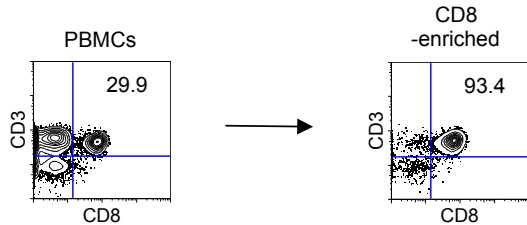**b**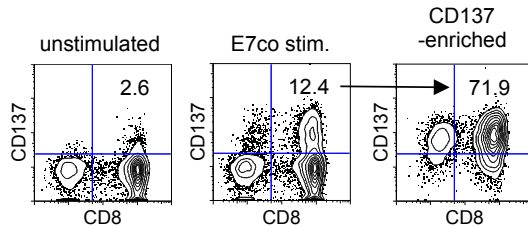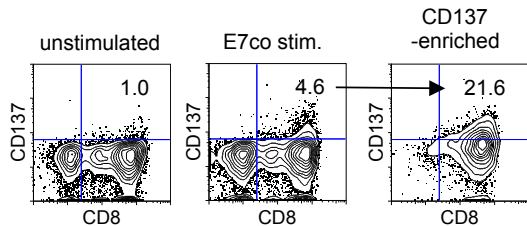

Supplement: S1 Fig — Data from two healthy donors are shown. (a) PBMCs were isolated from fresh blood via ficoll gradient centrifugation and enriched for CD8+ T cells by magnetic bead separation. Flow cytometry analysis of CD8+/CD3+ cells before and after CD8+ enrichment is shown. (b) After priming and expanding CD8+ T cells were stimulated with E7co-expressing DCs (E7co stim.) and sorted for CD137 activation marker via magnetic bead separation to obtain antigen-specific T cells (CD137-enriched). (PDF) [file pone.0121633.s001.pdf]

**a**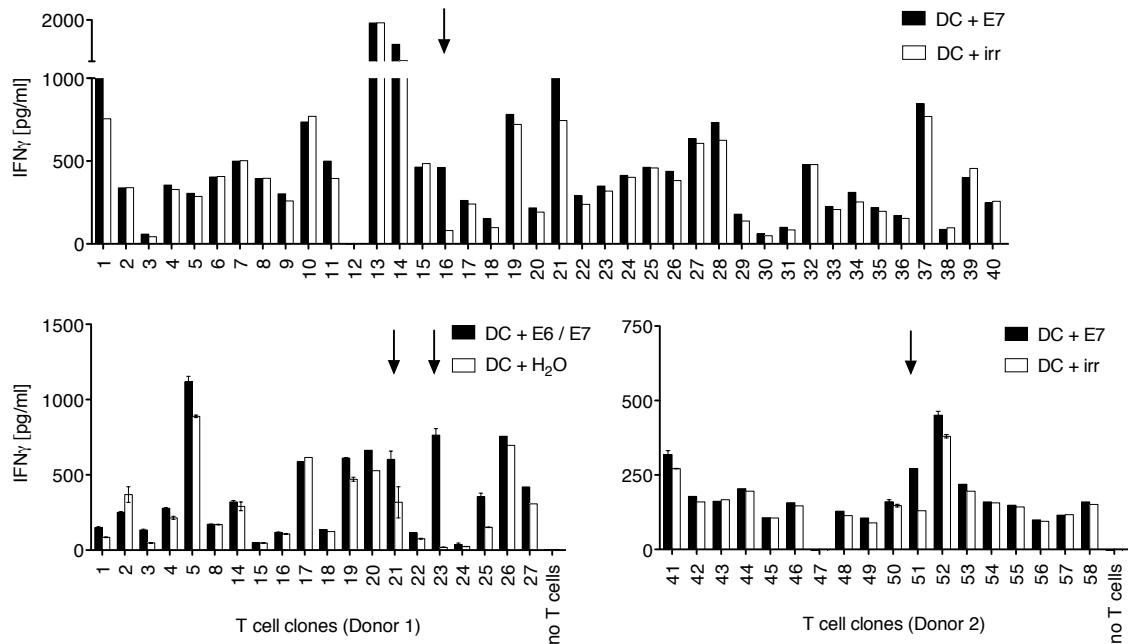**b**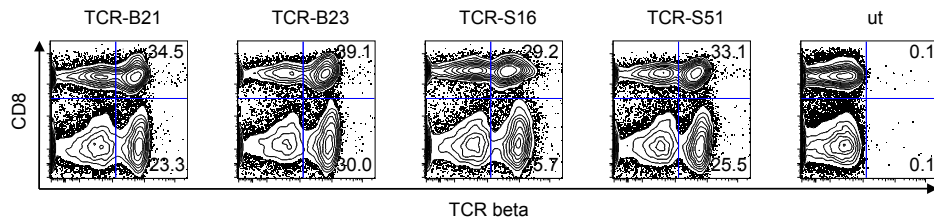

Supplement: S2 Fig — (a) T cell clones of both donors were cocultured with autologous DCs electroporated with or without E7co ivtRNA to identify antigen-specific T cells via IFNγ release. Arrows indicate selected antigen-specific T cell clones for isolation of TCR genes. Cocultures were performed in duplicates depending on the amount of cells available. Duplicates are shown as mean +/− SEM. (b) TCR genes of candidate T cell clones were isolated (S1 Table) and cloned with murine constant TCR regions into retroviral vectors for efficient expression of transgenic TCR to further analyze properties of TCR gene-modified T cells. Expression of transduced TCRs in T cells was detected by staining with an antibody specific for the murine constant beta region followed by flow cytometric analysis. Results are representative for 3 independent TCR transduction experiments. (PDF) [file pone.0121633.s002.pdf]

**a****E7co minigenes**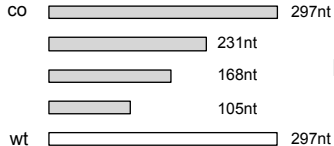**b**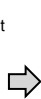

K562-B\*27:05

## TCR-B21

## TCR-B23

## TCR-S16

## TCR-S51

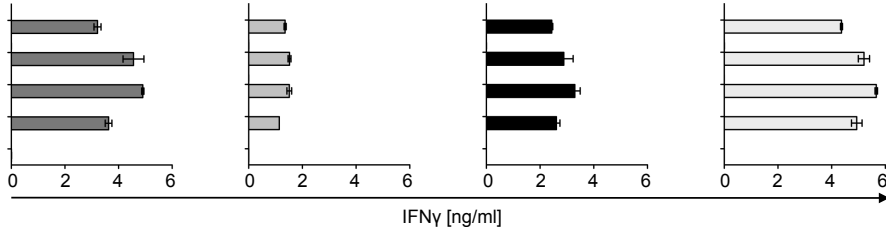

Supplement: S3 Fig — (a) Scheme of truncated minigenes of E7co for epitope mapping. Minigenes were stably expressed in K562-B*27:05 target cells via MP71 retrovirus transduction. Minigenes were coupled to mCherry expression marker via an IRES element to confirm transgene expression. (b) Supernatant of TCR-transduced T cells cocultured with target cells was screened for IFNγ release via ELISA. Results are shown as mean +/− SEM of duplicates. (PDF) [file pone.0121633.s003.pdf]
