# Supplementary material for: Codon Optimization of the Human Papillomavirus E7 Oncogene Induces a CD8+ T Cell Response to a Cryptic Epitope Not Harbored by Wild-Type E7
Source: PLoS One. 2015 Mar 23;10(3):e0121633. doi: 10.1371/journal.pone.0121633 (PMC4370481; doi:10.1371/journal.pone.0121633)
Supplement: S1 Table — TCR VDJ-gene usage was determined by PCR from cDNA of T cell clones with TCR chain specific primer panels. Resulting sequences were analyzed with IMGT/V-quest. TRAV, T cell receptor alpha variable region; TRBV, T cell receptor beta variable region; CDR3, complementary determining region 3. (PDF) [file pone.0121633.s004.pdf]

T cell donor	TCR	V-gene	D-gene	J-gene	CDR3
1	B21	TRAV14/DV4*01		TRAJ11*01	CAMRDWSAGYSTLTF
		TRBV27*01	TRBD1*01	TRBJ1-4*01	CASSLQGQLEEKLFF
1	B23	TRAV24*01		TRAJ24*02	CAHPTDSWGKLQF
		TRBV5-4*01	TRBD1*01	TRBJ2-1*01	CASSLALDNSYNEQFF
2	S16	TRAV8-4*03		TRAJ24*01	CAVYSWGKFQF
		TRBV28*01	TRBD1*01	TRBJ2-1*01	CASRPDSSSYNEQFF
2	S51	TRAV12-2*02		TRAJ50*01	CAVKSWETSYDKVIF
		TRBV6-5*01	TRBD2*01	TRBJ2-2*01	CASSYSGGLAGNTGELFF
